# Association between thyroid feedback quartile index and FIB-4-defined fibrosis risk in patients with metabolic dysfunction-associated steatotic liver disease, type 2 diabetes mellitus, and treated primary hypothyroidism: an exploratory cross-sectional study

**DOI:** 10.3389/fendo.2026.1875827

**Published:** 2026-08-10

**Authors:** Yuhan Wang, Jicheng Xie, Yan Wang, Xingning Liu, Xiaozhou Zhou

**Affiliations:** 1 Department of Liver Disease, The Fourth Clinical Medical College of Guangzhou University of Chinese Medicine, Shenzhen, Guangdong, China; 2 Department of Liver Disease, Shenzhen Traditional Chinese Medicine Hospital, Shenzhen, Guangdong, China; 3 Computer Science, WUYI University, Jiangmen, Guangdong, China; 4 Department of Emergency, Shenzhen Traditional Chinese Medicine Hospital, Shenzhen, Guangdong, China

**Keywords:** cross-sectional study, FIB-4, FIB-4-defined fibrosis risk, insulin resistance, metabolic dysfunction-associated steatotic liver disease, thyroid feedback quantile index, treated primary hypothyroidism, type 2 diabetes mellitus

## Abstract

**Background:**

Metabolic Dysfunction-associated Steatotic Liver Disease (MASLD) frequently coexists with Type 2 Diabetes Mellitus (T2DM) and Primary Hypothyroidism (PH); nevertheless, the relationship between thyroid hormone sensitivity and FIB-4-defined fibrosis risk across these metabolic diseases is yet unknown.

**Methods:**

In 102 patients with MASLD, MASLD+T2DM, and MASLD+T2DM+treated PH (34 per group), this exploratory cross-sectional study examined the association between the Thyroid Feedback Quantile Index (TFQI) and the Fibrosis-4 Index (FIB-4). The cumulative distribution functions of TSH and FT4 were used to calculate TFQI. A surrogate indicator of the risk of fibrosis was FIB-4. G*Power 3.1.9.7 was used for *post-hoc* power analysis after multivariable regression and correlation analyses were completed.

**Results:**

In the MASLD-alone group, TFQI and FIB-4 had a significant inverse correlation in unadjusted analysis (*r* = −0.384, *P* = 0.025). Nevertheless, after controlling for age, sex, and BMI, this link was diminished and no longer significant (*β* = −0.195, *P* = 0.073). The most powerful independent predictor of FIB-4 was age (*β* = 0.704, *P* < 0.001). Age significantly confounded the association, according to sensitivity analyses such as age-adjusted partial correlation, FIB-4 component analysis, and FIB-4 category analysis. In the other two groups, no noteworthy correlations were found.

**Conclusions:**

Age played a major role in explaining the inverse correlation between TFQI and FIB-4 in patients with MASLD alone in this exploratory study. These results should be considered hypothesis-generating and should be validated in bigger prospective trials with direct measurements of fibrosis. Future research may benefit from the heterogeneous associations across metabolic backgrounds.

## 1 Introduction

About 30% of people worldwide suffer from Metabolic Dysfunction-associated Steatotic Liver Illness (MASLD), the most prevalent chronic liver disease (1). MASLD can progress to hepatocellular carcinoma, cirrhosis, and end-stage liver disease, all of which have a significant impact on patients’ lives and long-term survival (2, 3). It is also strongly associated with increased risks of chronic renal disease, cardiovascular disease, and extrahepatic malignant tumors. Type 2 Diabetes Mellitus (T2DM) and Primary Hypothyroidism (PH) are two metabolic conditions that frequently coexist with MASLD. The risk of illness development and unfavorable outcomes is increased by this intricate web of co-morbidities (4, 5). Roughly 2.5% of MASLD patients have PH, and up to 28% have T2DM (6, 7). The substantial clustering of these metabolic diseases suggests a common pathophysiologic etiology, such as insulin resistance, chronic inflammation, and endocrine dysfunction (8).

Hepatic lipid metabolism, glucose homeostasis, and energy expenditure are all regulated by the thyroid gland (9). Epidemiological research indicates that both overt and subclinical hypothyroidism are associated with a higher incidence of MASLD and more severe liver histopathological characteristics (10, 11). Experiments have demonstrated that elevated thyrotropin levels in hypothyroidism directly activate hepatic stellate cells, promote collagen deposition, and accelerate hepatic fibrosis (12).However, most current studies concentrate on thyroid hormone levels, and little is known about the relationship between thyroid hormone sensitivity and FIB-4-defined fibrosis risk, or how responsive tissues are to thyroid hormones. The hypothalamic-pituitary-thyroid axis’s negative feedback regulation keeps thyroid hormone levels within a somewhat narrow physiological range, and many individuals experience metabolic issues even when their thyroid function markers are normal. Furthermore, in patients with hypothyroidism, exogenous thyroid hormone therapy (such as levothyroxine) can restore thyroid function, but tissue sensitivity might not be completely restored (13). Therefore, evaluating thyroid hormone sensitivity instead of only hormone levels could provide more insight into its relationship to hepatic fibrosis.

A new measure for evaluating central thyroid hormone sensitivity is the Thyroid Feedback Quantile-based Index (TFQI). It is computed using the cumulative distribution functions (*CDFs*) of TSH and FT4 in a reference population. By integrating the distribution positions of FT4 and TSH, the index measures the degree to which the pituitary feedback response to thyroid hormones deviates from the normal median level. This reflects the feedback status of the hypothalamic-pituitary-thyroid axis and captures subtle functional abnormalities, such as thyroid hormone resistance or hypersensitivity (14).Obese mice showed a reduced thyrotropic response to thyrotropin-releasing hormone stimulation, according to Wang et al. (15). Additionally, clinical research has demonstrated a strong correlation between TFQI and hepatic fibrosis. According to the NHANES data from 4,678 Americans with normal thyroid function, elevated TFQI was associated with increased fibrosis risk (16). Nevertheless, the majority of these investigations have only included individuals with normal thyroid function, and little attention has been given to patients with T2DM or PH—particularly those who have achieved treatment targets—nor have the effects of different metabolic backgrounds on the relationship between TFQI and FIB-4-defined fibrosis risk been compared in the framework of the same study.

In order to examine the relationship between central thyroid hormone sensitivity (TFQI) and FIB-4-defined fibrosis risk, we used a three-group design in this exploratory study: MASLD alone, MASLD with T2DM, and MASLD with T2DM and treated PH. Given that age is a factor in the FIB-4 computation, we sought to determine if the relationship between TFQI and FIB-4 varies across distinct metabolic scenarios, paying special attention to the potential confounding impact of age. Additionally, we looked at a number of secondary exploratory markers, such as glycemic parameters, ferritin, and insulin resistance. Instead of being confirming, all analyses are meant to generate hypotheses.

## 2 Methods

### 2.1 Study design and population

This was a single-center retrospective cross-sectional study. Consecutive patients who attended Shenzhen Hospital of Traditional Chinese Medicine from March 2021 to March 2026 were enrolled. A total of 102 patients were finally included and divided into three groups:

Group A (triple-disease group, *n* = 34): patients who met the diagnoses of MASLD, T2DM, and PH at the same time, were receiving levothyroxine replacement therapy, and had thyroid function (TSH, FT4) within the normal range over the preceding 3 months.

Group B (two-disease group, *n* = 34): patients who met the diagnoses of MASLD and T2DM, had consistently normal thyroid function (no history of hypothyroidism; TSH and FT4 within normal range).

Group C (control group, *n* = 34): patients with MASLD, without T2DM, and with consistently normal thyroid function.

### 2.2 Inclusion and exclusion criteria

#### 2.2.1 Inclusion criteria

①Age between 18 and 80 years (inclusive); no sex restriction.

②Diagnosis of MASLD: according to the 2023 International Multi-Society Delphi Consensus’ definition (17). Patients had to fulfill each of these requirements: (a) hepatic steatosis confirmed by abdominal ultrasound or transient elastography; and (b) at least one cardiometabolic risk factor, such as dyslipidemia, hypertension, T2DM, or overweight/obesity.

③Further categorized into the following three groups based on disease status:

Group A (triple diagnosis group): patients concurrently meeting the diagnoses of MASLD, T2DM and PH. T2DM was defined according to ADA criteria (18): fasting glucos*e* ≥ 7.0 mmol/L, HbA1c ≥ 6.5%, OGTT 2h blood glucose ≥ 11.1 mmol/L, random glucose ≥ 11.1 mmol/L with hyperglycemic symptoms, or a documented history of T2DM with glucose-lowering therapy.PH was defined by a clear diagnosis and current levothyroxine replacement therapy, with TSH and FT4 within normal limits over the preceding 3 months.

Group B (dual-disease group): patients with MASLD and T2DM, with consistently normal thyroid function (no history of PH; TSH, FT4, and FT3 within normal ranges over the preceding 3 months).

Group C (MASLD-only control group): patients with MASLD, without T2DM, and with consistently normal thyroid function. T2DM was excluded by fasting glucose < 7.0 mmol/L, HbA1c < 6.5%, no history of T2DM, and no glucose-lowering therapy (18).

④Complete clinical data were required, including sex, age, height, weight, BMI, liver and kidney function, blood lipids, blood glucose, thyroid function, complete blood count and abdominal ultrasound results.

#### 2.2.2 Exclusion criteria

①Liver diseases caused by other factors: alcoholic liver disease (ethanol intake > 30 g/day for men and > 20 g/day for women within the past year (19)), chronic viral hepatitis, drug-induced liver injury, cirrhosis, etc.

②Severe systemic diseases: including malignant tumors; hematological disorders; and autoimmune diseases (psoriasis, systemic lupus erythematosus, etc.).

③Hypothalamic-pituitary disorders: pituitary adenomas, craniopharyngiomas, central hypothyroidism, etc.

④Severe renal impairment.

⑤Recent (within 3 months) use of medications that may significantly affect metabolism: glucocorticoids, antipsychotic drugs (olanzapine, clozapine, quetiapine, etc.).

⑥Pregnant or breastfeeding women.

### 2.3 Definition and calculation of indexes

①Thyroid feedback quantile index *(*TFQI):


$$TFQI={\text{CDF}}_{FT4}-(1-{\text{CDF}}_{TSH})$$

with a range of values from -1 to 1, and lesser central thyroid hormone sensitivity is indicated by greater readings (14).

②Hepatic fibrosis index (FIB-4):


$$FIB-4=\frac{age\times AST(\text{U}/\text{L})}{platelet(\times 10^{9}/\text{L})\times \sqrt{\text{ALT}(\text{U}/\text{L})}}\;$$

(20)③APRI:


$$APRI=\frac{AST(\text{U}/\text{L})/40}{platelet(\times 10^{9}/\text{L})}\times 100$$

(21)④TyG index:


$$TyG=\ln(\frac{TG(\text{mg}/\text{dL})\times \text{FBG}(\text{mg}/\text{dL})}{2})$$

In units of mmol/L, triglycerides × 88.57 and fasting glucose × 18.02 were used for conversion (22).

⑤*Ferritin*: due to skewed distribution (Shapiro-Wilk test, *P* = 0.009), natural logarithmic transformation (log_ferritin) was applied before analysis.

⑥Insulin resistance: *fasting C-peptide* (natural log-transformed) was used as a surrogate marker.

### 2.4 Statistical analysis

SPSS 31.0 was used for statistical analysis. The Shapiro-Wilk test was used to determine whether continuous variables were normal. Mean ± standard deviation (Mean ± SD) is used to express variables with a normal distribution. One-way analysis of variance (ANOVA) was used to compare several groups, and LSD *post-hoc* tests were used for pairwise comparisons. The Kruskal–Wallis H test was employed for multi-group comparisons, and those who did not fit the normal distribution were displayed as median (interquartile range) [M(Q1,Q3)]. Frequency (percentage) [*n*(%)] was used to indicate count data, and Fisher’s exact test or the χ² test were used to compare groups. Depending on the distribution of the data, Pearson or Spearman correlation coefficients were utilized in correlation studies. Confounders were adjusted for using multiple linear regression, and mediation analysis was conducted using manual three-step regression. *P* < 0.05 was regarded as statistically significant, and all tests were two-sided.

This study was a retrospective analysis, and the sample size was determined by consecutive cases that met the inclusion and exclusion criteria. A *post-hoc* statistical power analysis (*α* = 0.05, two-sided test) for significant correlations was performed using G*Power 3.1.9.7. The study demonstrated that statistical power was limited in all groups due to the relatively small sample size (34 patients per group). Group C’s observed correlation (*r* = −0.384, *n* = 34) yielded a power of 0.63, which is slightly below the conventional cutoff of 0.80 but acceptable in an exploratory retrospective study. With a power of 0.40 (*r* = 0.296, *n* = 32), Group B had insufficient statistical power to detect the trend. Therefore, non-significant or trend-level data should be viewed as exploratory and hypothesis-generating rather than final. In Group A, no significant correlation was observed and the effect size was small, so *post-hoc* power was not calculated.

The connection between TFQI and FIB-4 in each of the three groups served as the primary analysis. In Group C, multivariable linear regression was used to account for potential confounders (age, sex, and BMI). The robustness of the main findings was evaluated using sensitivity analyses, such as FIB-4 component analysis, age-adjusted partial correlation, and FIB-4 category analysis. The remaining analyses (APRI, TyG index, ferritin, HbA1c, liver enzymes, and mediation analysis) are described as exploratory.

## 3 Results

### 3.1 Baseline characteristics

**Figure 1:**
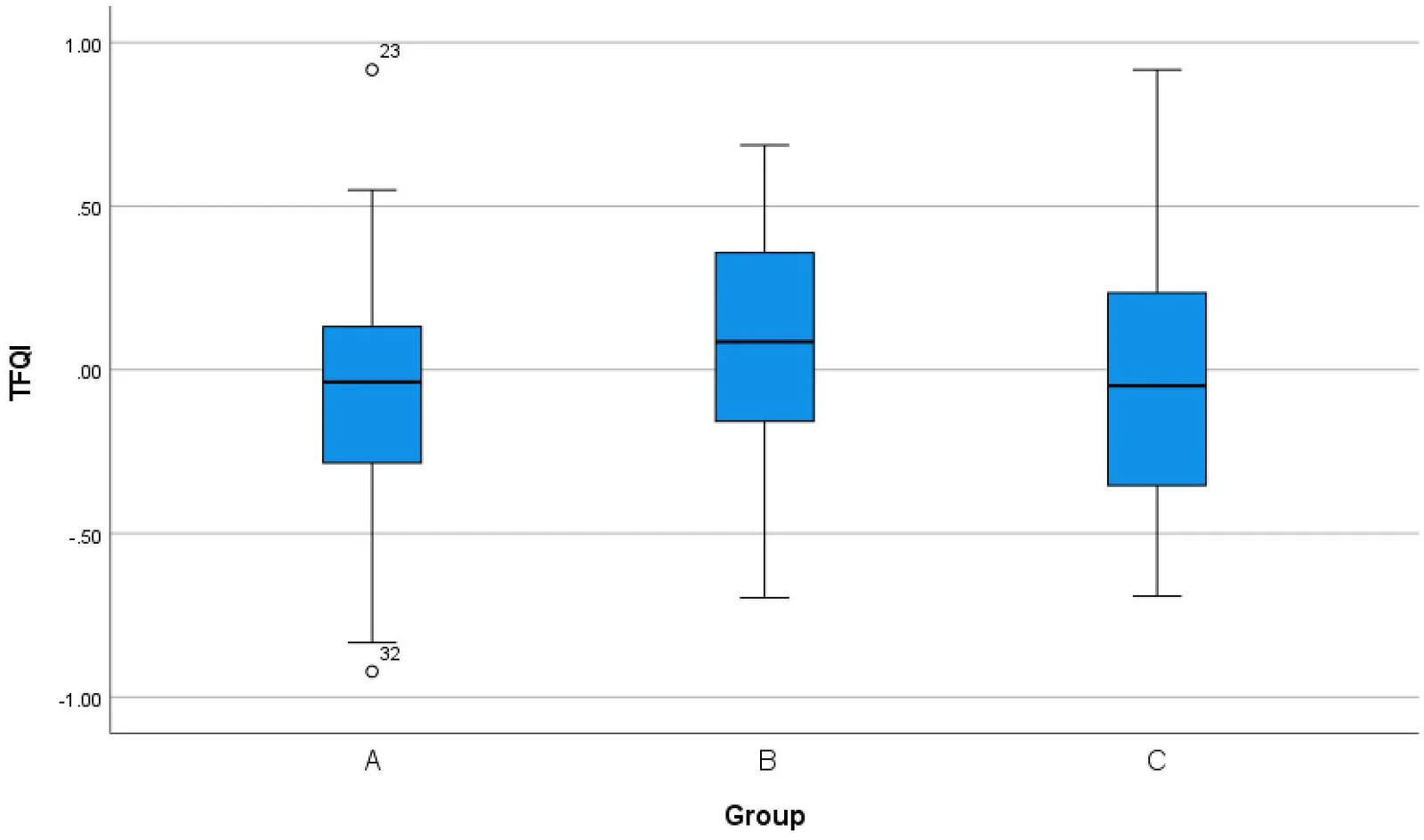
Box plot of TFQI levels in the three groups (A:MASLD+T2DM+treated hypothyroidism, *n* = 34; B:MASLD+T2DM, *n* = 34; C:MASLD alone, *n* = 34). *P* = 0.285 (one-way ANOVA).

This study included 102 patients (34 in each group; baseline characteristics are shown in **Table 1**). Age and sex distribution differed significantly among the three groups (*P* < 0.001 and *P* = 0.003): Group A had the oldest patients, whereas Group C had the highest proportion of males. Groups A and B had considerably higher fasting glucose and HbA1c values (both *P* < 0.001) than Group C, which is consistent with the clinical features of the diabetic groupings. The three groups’ FIB-4 levels varied significantly (*P* < 0.001), with Group A having the highest levels and Groups B and C having comparable amounts. In terms of thyroid function, Group C had the highest FT3 level while Group A had the highest TSH level (both *P* < 0.001). There was no discernible variation in the three groups’ TFQI levels (*P* = 0.285, **Figure 1**).

**Table 1:** Baseline characteristics of the three groups.

Variable	Group A(*n* = 34)	Group B(*n* = 34)	Group C(*n* = 34)	*P*-value
Demographic characteristics				
Age (years)	60.65 ± 8.48	47.38 ± 15.09	46.71 ± 10.75	<0.001^*^
Male, *n*(%)	11 (32.4)	18 (52.9)	25 (73.5)	0.003^†^
BMI (kg/m²)	25.78 ± 2.97	27.54 ± 3.86	27.16 ± 3.02	0.075^‡^
Glycemic parameters				
HbA1c (%)	7.20 (6.65, 8.70)	8.60 (7.30, 9.45)	5.75 (5.60, 5.90)	<0.001^§^
Fasting glucose (mmol/L)	6.25 (5.67, 8.13)	8.67 (6.04, 10.31)	5.10 (4.88, 5.35)	<0.001^§^
Thyroid-related parameters				
TSH (pmol/L)	3.39 (2.05, 4.55)	1.85 (1.38, 2.44)	1.93 (1.34, 2.57)	<0.001^§^
FT4 (pmol/L)	12.25(10.60, 12.90)	11.45 (10.80, 12.40)	11.60 (10.00, 12.50)	0.141^§^
FT3 (pmol/L)	4.45 (4.10, 4.87)	5.31 (4.79, 5.76)	5.38 (5.02, 6.02)	<0.001^§^
TFQI	-0.087 ± 0.15	0.067 ± 0.18	0.020 ± 0.14	0.285^‡^
Liver function				
ALT (U/L)	18.65 (13.90, 28.70)	23.45 (16.70, 37.10)	27.70 (18.10, 59.80)	0.182^§^
AST (U/L)	18.30 (14.80, 27.00)	18.25 (15.40, 25.30)	21.65 (17.60, 31.50)	0.122^§^
GGT (U/L)	23.0 (18.0, 37.3)	25.0 (17.6, 40.2)	53.9 (21.6, 105.5)	0.008^§^
FIB-4	1.26 (0.78, 1.62)	0.69 (0.48, 1.08)	0.76 (0.54, 0.99)	<0.001^§^
Lipid profile				
*Triglycerides* (mmol/L)	1.85 (1.44, 3.30)	1.81 (1.37, 2.42)	1.82 (1.02, 2.29)	0.391^§^
Total cholesterol (mmol/L)	4.45 (3.61, 5.00)	4.85 (4.37, 5.45)	5.07 (4.36, 5.57)	0.013^§^
*HDL-C* (mmol/L)	1.05 ± 0.27	1.01 ± 0.23	1.17 ± 0.37	0.072^‡^
LDL-C (mmol/L)	2.56 ± 0.92	3.07 ± 0.84	3.16 ± 0.79	0.010^‡^
Renal function				
eGFR (mL/min/1.73 m²)	95.56 ± 12.44	105.88 ± 17.72	100.47 ± 10.03	0.010^‡^
Complete blood count				
Platelet count (×10^9^/L)	228.12 ± 64.96	251.18 ± 58.12	270.32 ± 54.14	0.016^‡^

Data are expressed as mean ± SD for normally distributed variables, median (Q1, Q3) for skewed variables, and *n* (%) for categorical variables. The number of available cases for each variable is as follows: HbA1c, *n* = 31 in Group A and Group B, *n* = 34 in Group C; *fasting glucose*, *n* = 33 in Group A, *n* = 34 in Group B and Group C; FIB-4, *n* = 33 in Group B, *n* = 34 in Group A and Group C; *total cholesterol*, *triglycerides*, *HDL-C*, and *LDL-C*, *n* = 33 in Group A and Group B, *n* = 34 in Group C; *platelet count*, *n* = 33 in Group B, *n* = 34 in Group A and Group C. All other variables had complete data (*n* = 34 per group).

P-values:.

† Chi-square test;.

‡ One-way ANOVA (with *post-hoc* LSD or Tamhane’s T2 when applicable);.

§ Kruskal–Wallis test (with *post-hoc* Mann–Whitney U test and Bonferroni correction when applicable).

*Post-hoc* pairwise comparisons (only for variables with overall *P* < 0.05):.

Age (ANOVA, LSD): A vs. B and A vs. C, both *P* < 0.001.

HbA1c (Kruskal–Wallis, Mann–Whitney with Bonferroni): A vs. C and B vs. C, both *P* < 0.001; A vs. B, *P* = 0.092 (ns after correction).

Fasting glucose (Kruskal–Wallis, Mann–Whitney with Bonferroni): A vs. B, *P* = 0.008; A vs. C and B vs. C, both *P* < 0.001.

TSH (Kruskal–Wallis, Mann–Whitney with Bonferroni): A vs. B and A vs. C, both *P* < 0.001; B vs. C, *P* = 0.859.

FT3 (Kruskal–Wallis, Mann–Whitney with Bonferroni): A vs. B and A vs. C, both *P* < 0.001; B vs. C, *P* = 0.121.

GGT (Kruskal–Wallis, Mann–Whitney with Bonferroni): A vs. C, *P* = 0.007; B vs. C, *P* = 0.008; A vs. B, *P* = 0.820.

FIB-4 (Kruskal–Wallis, Mann–Whitney with Bonferroni): A vs. B and A vs. C, both *P* < 0.001; B vs. C, *P* = 0.616.

Total cholesterol (Kruskal–Wallis, Mann–Whitney with Bonferroni): A vs. C, *P* = 0.005; A vs. B, *P* = 0.024 (ns after correction); B vs. C, *P* = 0.688.

LDL-C (ANOVA, LSD): A vs. B, *P* = 0.017; A vs. C, *P* = 0.005; B vs. C, *P* = 0.661.

eGFR (ANOVA, Tamhane’s T2 due to unequal variances): A vs. B, *P* = 0.022; A vs. C, *P* = 0.216; B vs. C, *P* = 0.335.

Platelet count (ANOVA, LSD): A vs. C, *P* = 0.004; A vs. B, *P* = 0.114; B vs. C, *P* = 0.189.

### 3.2 Correlation between TFQI and FIB-4

**Figure 2:**
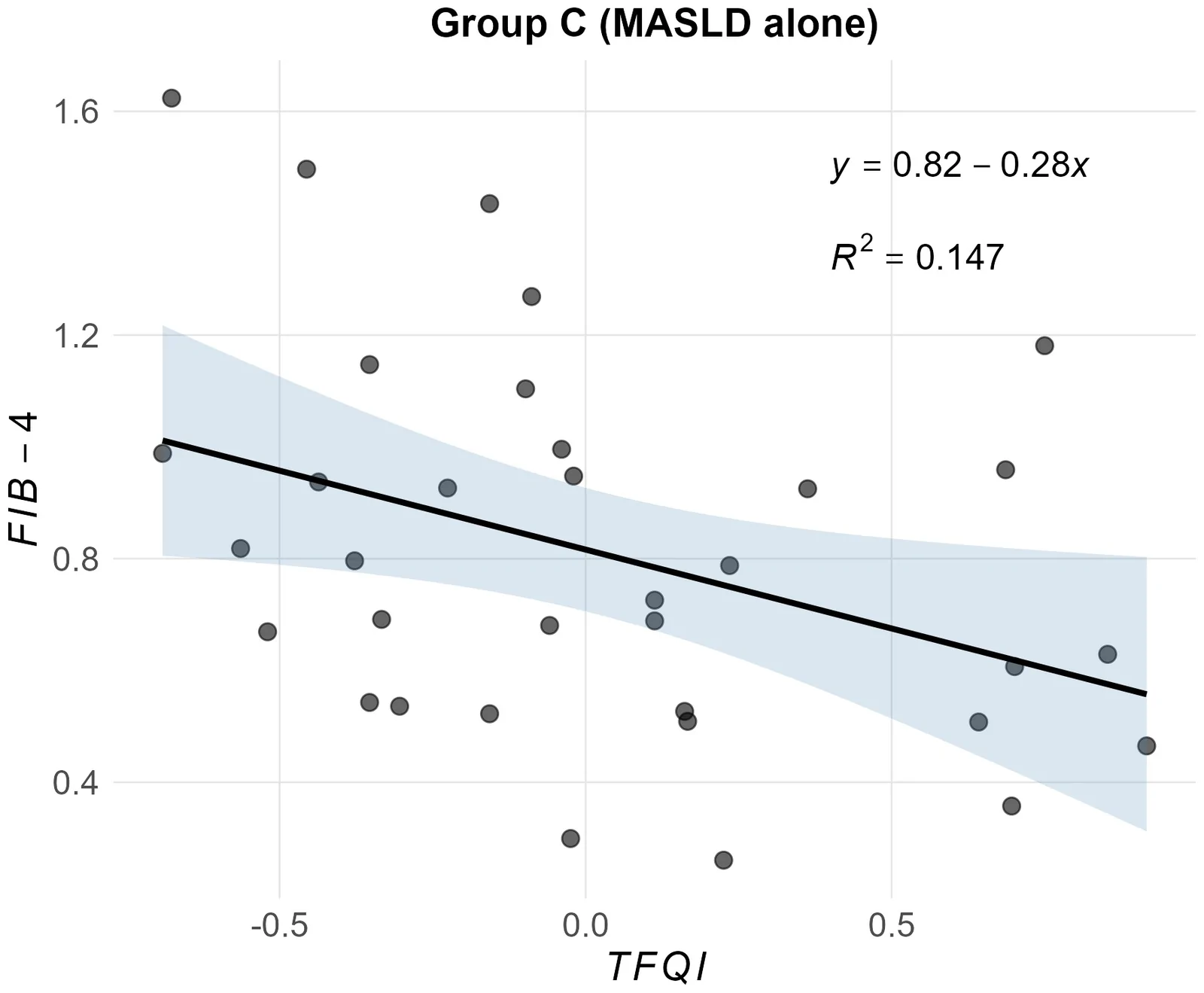
Scatter plot of TFQI vs FIB-4 in Group C (MASLD alone, *n* = 34). Shaded areas represent 95% confidence intervals for the regression line. *r* = −0.384, *P* = 0.025.

**Figure 3:**
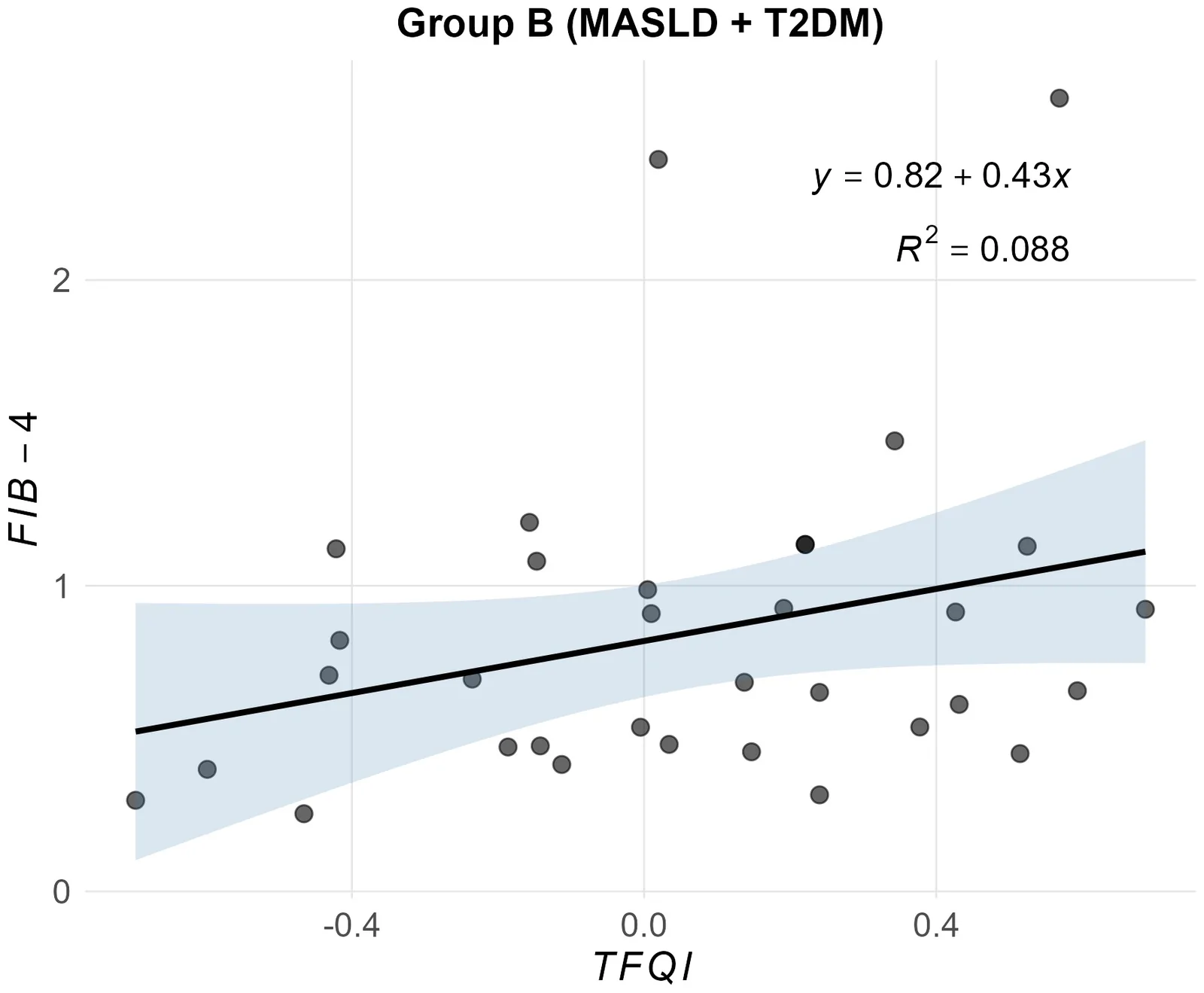
Scatter plot of TFQI vs FIB-4 in Group B (MASLD with T2DM, *n* = 33). One case was excluded due to missing platelet count. Shaded areas represent 95% confidence intervals for the regression line. *r* = 0.296, *P* = 0.094.

**Table 2** displays the findings of the Pearson correlation study between the three groups’ TFQI and FIB-4. TFQI and FIB-4 had a significant negative correlation in Group C (*n* = 34) (*r* = −0.384, *P* = 0.025, *y* = 0.82 − 0.28*x*, *R²* = 0.147, **Figure 2**). TFQI and FIB-4 exhibited a positive association trend in Group B (*n* = 33) (*r* = 0.296, *P* = 0.094, *y* = 0.82 + 0.43*x*, *R²* = 0.088, **Figure 3**). The two did not significantly correlate in Group A (*n* = 34) (*r* = 0.166, *P* = 0.350).

**Table 2:** Summary of correlations between TFQI and FIB-4.

Group	*n*	*r*	*P-value*	Regression equation	*R^2^*
A	34	0.166	0.350	—	—
B	33	0.296	0.094	*y* = 0.82 + 0.43*x*	0.088
C	34	−0.384	0.025	*y* = 0.82 − 0.28*x*	0.147

### 3.3 Multivariable linear regression for confounding factors

A straightforward correlation analysis between TFQI and FIB-4 in Group C revealed a significant negative association (*r* = −0.384, *P* = 0.025). Then, using FIB-4 as the dependent variable and TFQI, age, sex, and BMI as independent factors, a multivariable linear regression model was built. The model was statistically significant overall (adjusted *R^2^* = 0.694, *F* = 11.434, *P* < 0.001). The findings indicated that sex was marginally significant (*β* = 0.362, *P* = 0.066), age was the largest independent predictor of FIB-4 (standardized coefficient *β* = 0.704, *P* < 0.001), and BMI was not significantly connected with FIB-4 (*β* = −0.145, *P* = 0.337). After normalization, the negative correlation trend between TFQI and FIB-4 diminished (*β* = −0.195, *P* = 0.073, **Table 3**).

**Table 3:** Primary and sensitivity analyses for the association between TFQI and FIB-4 in Group C.

Analysis	Method	n	Effect estimate	95% CI/P-value
Primary analysis				
Unadjusted correlation	Spearman correlation	34	r = −0.384	P = 0.025
Multivariable regression	Linear regression	34		
TFQI			β = −0.195	P = 0.073
Age			β = 0.704	P < 0.001
Sex			β = 0.362	P = 0.066
BMI			β = −0.145	P = 0.337
Sensitivity analyses				
Age-adjusted partial correlation	Spearman partial correlation	34	partial r = −0.281	P = 0.114
FIB-4 component: Age	Spearman correlation	34	ρ = −0.324	P = 0.062
FIB-4 component: AST/ALT ratio	Spearman correlation	34	ρ = −0.059	P = 0.740
FIB-4 component: Platelet count	Spearman correlation	34	ρ = 0.332	P = 0.055
FIB-4 category analysis	Descriptive	34	Not estimable†	—

† FIB−4 risk categories: low (<1.30), intermediate (1.30–2.67), high (>2.67). In Group C (*n* = 34), 31 patients were in the low−risk category, 3 in the intermediate−risk category, and none in the high−risk category. Formal statistical comparison across categories was not feasible due to the highly imbalanced distribution and small sample size.

### 3.4 Sensitivity analyses

#### 3.4.1 FIB-4 component analysis

In Group C (*n* = 34), Spearman associations between TFQI and age, AST/ALT ratio, and platelet count were investigated. TFQI had no correlation with the AST/ALT ratio (ρ = −0.059, *P* = 0.740), a marginal negative correlation with age (ρ = −0.324, *P* = 0.062), and a marginal positive correlation with platelet count (ρ = 0.332, *P* = 0.055) (**Table 3**).

#### 3.4.2 Age-adjusted partial correlation analysis

After adjusting for age, a partial correlation study (Spearman partial correlation) was conducted between TFQI and FIB-4 in Group C. Compared to the unadjusted association (*r* = −0.384, *P* = 0.025), the partial correlation was weakened and no longer significant after age correction (partial *r* = −0.281, *P* = 0.114) (**Table 3**).

#### 3.4.3 FIB-4 category analysis

Based on known clinical cutoffs, patients were divided into three FIB-4 risk categories: low-risk (< 1.30), intermediate-risk (1.30–2.67), and high-risk (> 2.67). Only three patients were in the intermediate-risk group and none were in the high-risk group in each cohort, with over 90% of patients in Groups B and C falling into the low-risk category. Twenty-one patients in Group A were classified as low-risk, ten as intermediate-risk, and three as high-risk. Because of the short sample size and extremely unbalanced distribution, a formal statistical comparison of TFQI across FIB-4 risk groups was not possible (**Table 3**).

### 3.5 Exploratory analyses

#### 3.5.1 Correlation between TFQI and APRI

While there was no significant link between APRI and FIB-4 (*r* = 0.156, *P* = 0.378, **Table 4**), Pearson’s correlation analysis revealed a negative trend between TFQI and APRI (*r* = −0.213, *P* = 0.226) in Group C (*n* = 34).

**Table 4:** Summary of secondary and exploratory analyses.

Analysis	Group	*n*	Statistic	Value	*P-value*
Ferritin					
*log−ferritin* vs. FIB-4 (Pearson)	C	21	*r*	−0.333	0.140
*log−ferritin* for FIB-4 (adjusted for age, sex, BMI)	C	21	Standardized *β*	0.290	0.135
Alternative fibrosis marker					
TFQI vs. APRI (Spearman)	C	34	*ρ*	−0.213	0.226
APRI vs. FIB-4 (Pearson)	C	34	*r*	0.156	0.378
Peripheral thyroid sensitivity					
FT3*/*FT4 ratio vs. FIB-4 (Pearson)	C	34	*r*	0.141	0.427
Glycemic control					
HbA1c vs. FIB-4 (Pearson)	B	30	*r*	0.044	0.817
Interaction TFQI × HbA1c for FIB-4	C	34	Standardized *β*	1.339	0.795
Liver enzymes (Spearman)					
TFQI vs. ALT	C	34	*ρ*	0.082	0.646
TFQI vs. AST	C	34	*ρ*	0.025	0.890
TFQI vs. GGT	C	34	*ρ*	0.129	0.467
Insulin resistance marker (TyG index vs. FIB-4, Spearman)					
	A	32	*ρ*	−0.245	0.177
	B	33	*ρ*	−0.153	0.402
	C	34	*ρ*	−0.418	0.014
Mediation analysis (*fasting C-peptide*)					
TFQI → *C-peptide* (path a)	B	32	B (unstandardized)	0.013	0.963
*C-peptide* → FIB-4 (path b)	B	31	B (unstandardized)	−0.196	0.263

FIB-4, *fibrosis-4* index; TFQI, thyroid feedback quantile index; APRI, AST-to-platelet count ratio index; FT3, free triiodothyronine; FT4, free thyroxine; HbA1c, glycated hemoglobin; ALT, alanine aminotransferase; AST, aspartate aminotransferase; GGT, gamma-glutamyl transferase; TyG, triglyceride-glucose index.

All analyses are exploratory; *P-values* < 0.05 are considered indicative of statistical significance without adjustment for multiple comparisons.

#### 3.5.2 Correlation between TyG index and FIB-4

**Figure 4:**
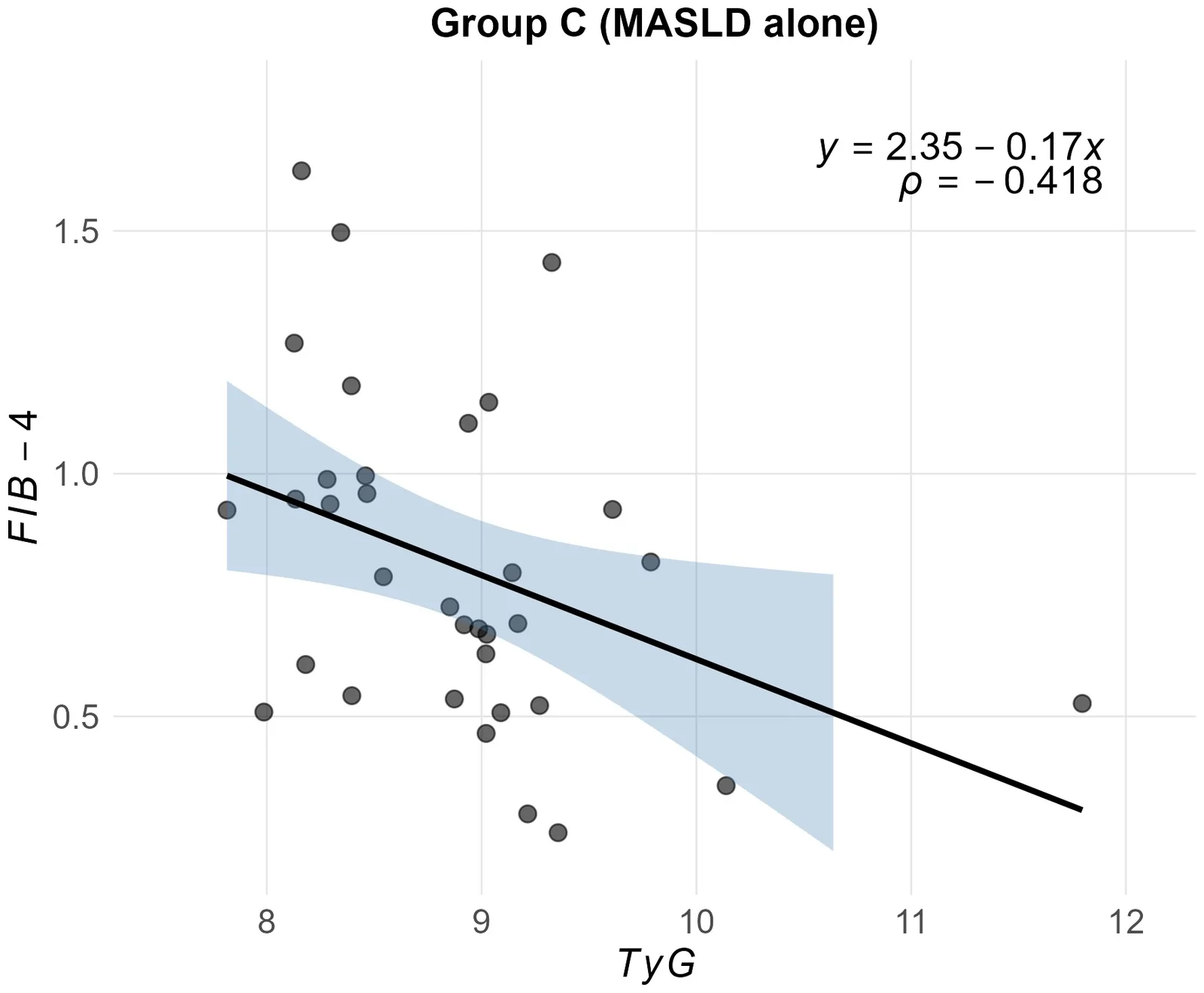
Scatter plot of TyG index vs FIB-4 in Group C (MASLD alone, *n* = 34). Shaded areas represent 95% confidence intervals for the regression line. Spearman *ρ* = −0.418, *P* = 0.014.

The correlations between TyG and FIB-4 are shown in **Table 4**. A significant negative correlation was observed in Group C (*n* = 34) (*ρ* = −0.418, *P* = 0.014; **Figure 4**), whereas no significant correlation was found in Group A (*n* = 32, *ρ* = −0.245, *P* = 0.177) or Group B (*n* = 33, *ρ* = −0.153, *P* = 0.402).

#### 3.5.3 Comparison and correlation of HbA1c

**Figure 5:**
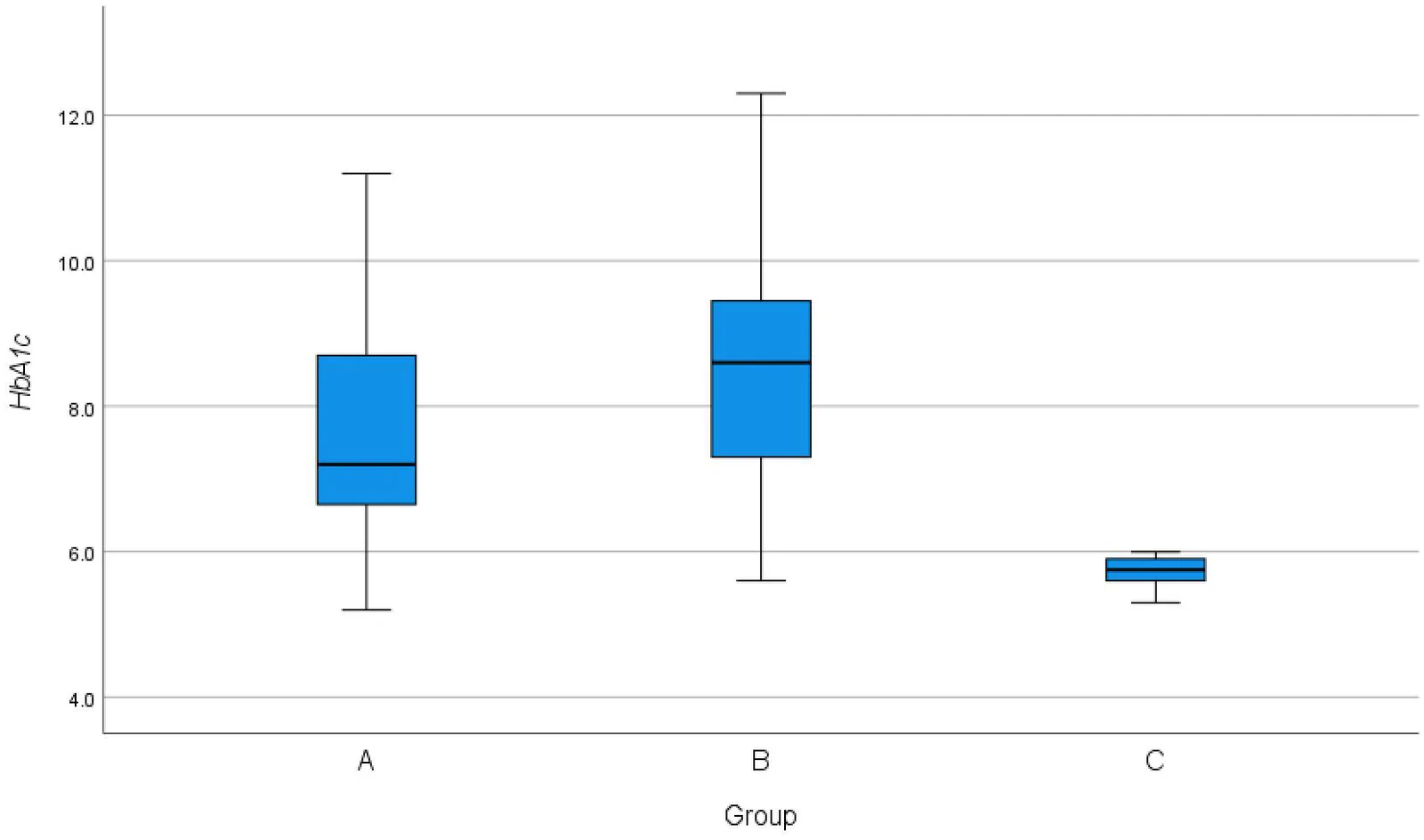
Box plot of HbA1c levels in the three groups. *P* < 0.001 (one-way ANOVA with Tamhane’s T2 *post-hoc*).

The three groups’ HbA1c levels varied significantly (*F* = 31.683, *P* < 0.001), with Groups A and B having higher values than Group C (both *P* < 0.001, **Figure 5**). In Group B, there was no significant association between HbA1c and FIB-4 (*r* = 0.044, *P* = 0.817), while in Group C, there was no significant interaction between HbA1c and TFQI on FIB-4 (*β* = 1.339, *P* = 0.795, **Table 4**).

#### 3.5.4 FT3*/*FT4 ratio and FIB-4 correlation

The FT3*/*FT4 ratio did not significantly correlate with FIB-4 in Group C (*n* = 34, *r* = 0.141, *P* = 0.427; **Table 4**).

#### 3.5.5 Correlation between ferritin and FIB-4

**Figure 6:**
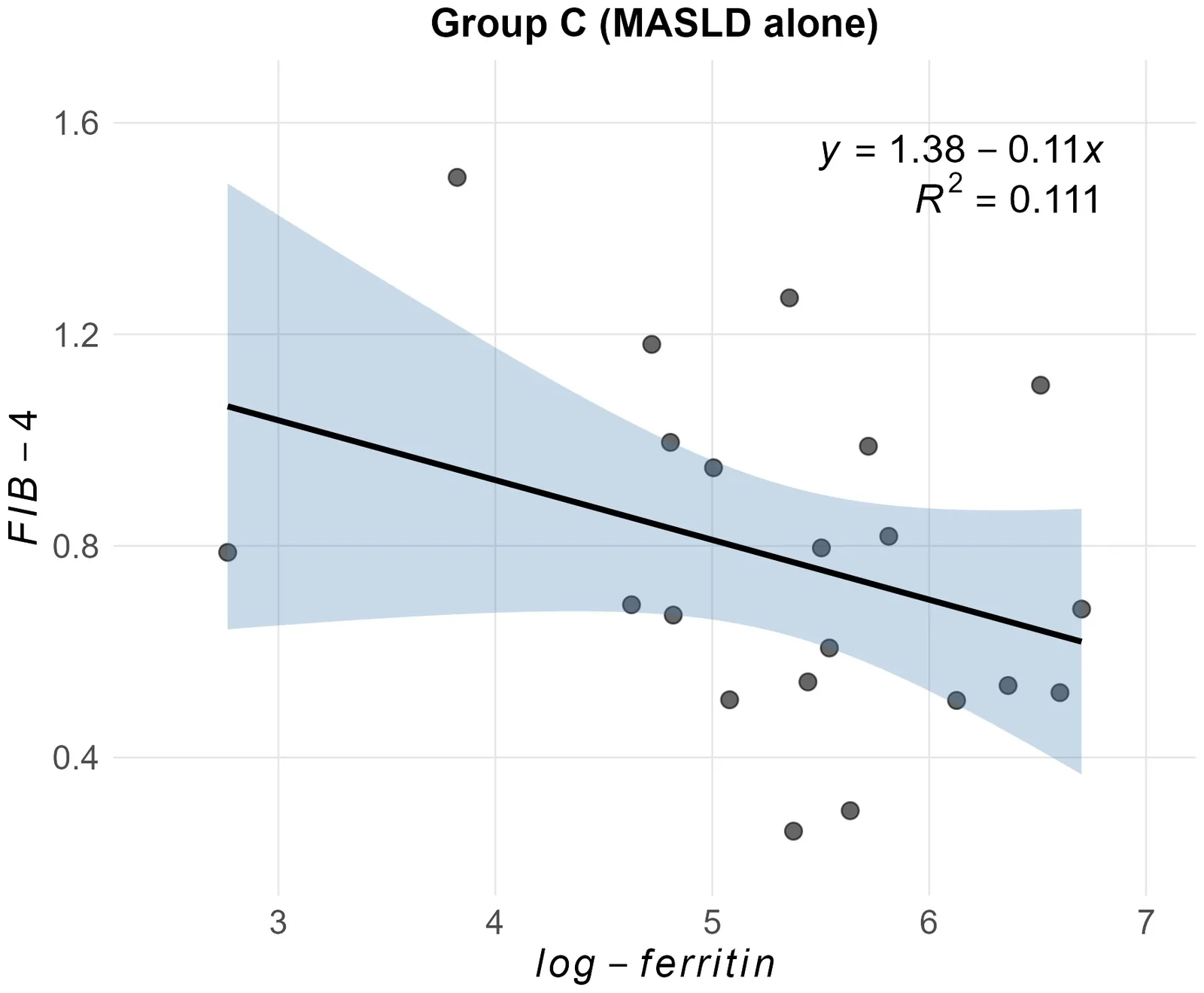
Scatter plot of *log-ferritin* vs FIB-4 in Group C (MASLD alone, *n* = 21). Shaded areas represent 95% confidence intervals for the regression line. *r* = −0.333, *P* = 0.140.

In Group C, log−ferritin showed a negative correlation trend with FIB-4 (*n* = 21, *r* = −0.333, *P* = 0.140, **Figure 6**); however, it was not independently associated with FIB-4 after adjustment for age, sex, and BMI (*β* = 0.290, *P* = 0.135, **Table 4**).

#### 3.5.6 Correlation between liver enzymes and TFQI

In Group C, TFQI was not significantly associated with ALT (*ρ* = 0.082, *P* = 0.646), AST (*ρ* = 0.025, *P* = 0.890), or GGT (*ρ* = 0.129, *P* = 0.467; **Table 4**) (Spearman).

### 3.6 Mediation analysis

In Group B, *fasting C-peptide* was used as a surrogate index of insulin resistance to test its mediating effect between TFQI and FIB-4 (natural log-transformed due to skewed distribution). Linear regression showed that TFQI had no significant effect on *ln_Cpeptide* (*n* = 32, *β* = 0.013, *P* = 0.963), and FIB-4 was not significantly affected by *ln_Cpeptide*.(*n* = 31,*β* = −0.196,*P* = 0.263, **Table 4**). Accordingly, no mediating effect was established.

## 4 Discussion

We examined the relationship between TFQI and FIB-4-defined fibrosis risk in three metabolic backgrounds (MASLD alone, MASLD+T2DM, and MASLD+T2DM+treated PH) in this exploratory study. We also looked at a number of secondary exploratory markers, such as ferritin, insulin resistance, and glycemic parameters.

Li et al. (23) observed a positive correlation between TFQI and hepatic steatosis (CAP) in euthyroid individuals, whereas Zhang et al. (24) discovered an inverse correlation between TFQI and advanced fibrosis (FIB-4) in diabetic patients. In a general euthyroid population, Yao et al. (16) found that greater TFQI was linked to an increased incidence of fibrosis; however, our findings in MASLD patients revealed an inverse correlation that was primarily explained by age. By enrolling individuals with treated PH and explicitly comparing three metabolic scenarios within a single study design, our work expands on these findings.

The key finding of our study is that, in patients with pure MASLD (Group C), the negative relationship between TFQI and FIB-4 was significant in unadjusted analysis (*r* = −0.384, *P* = 0.025), but it was weakened and no longer significant after adjusting for age, sex, and BMI (*β* = −0.195, *P* = 0.073). Sensitivity tests further supported this finding: TFQI revealed marginal associations with both age (ρ = −0.324, *P* = 0.062) and platelet count (ρ = 0.332, *P* = 0.055), two components of the FIB-4 index; age-adjusted partial correlation was not significant (partial *r* = −0.281, *P* = 0.114). These findings suggest that a significant confounding factor in the TFQI-FIB-4 association is age.

Notably, there was a difference in the direction of TFQI-FIB-4 relationships between Group B (positive, although not significant, *r* = 0.296, *P* = 0.094) and Group C (negative). Age was a major factor in explaining the inverse correlation in patients with pure MASLD; however, in patients with concurrent T2DM, the relationship tended in the reverse direction. Instead of a straightforward additive effect of two metabolic disorders, this reversal implies that the presence of diabetes may create metabolic interactions that alter the thyroid-liver relationship. Further evidence that adequate levothyroxine replacement may restore central thyroid feedback to the point where peripheral effects on the liver become undetectable in this sample comes from the lack of any correlation in patients with treated PH (Group A, *r* = 0.166, *P* = 0.350). The main discovery of this exploratory work is that these phenotypic variations among metabolic states—rather than a single, cohesive pathway—call for additional research in more powerful studies.

Age was the most powerful independent predictor of FIB-4 in multivariable regression (*β* = 0.704, *P* < 0.001), and patients in Group A were significantly older than those in Groups B and C (both *P* < 0.001). The natural history of primary hypothyroidism, which is more common in older populations, probably accounts for this age difference. Previous research has repeatedly shown that age is a significant risk factor for the development of fibrosis (25).The greater FIB-4 levels shown in Group A may be due to age rather than more severe fibrosis because age is a factor in the FIB-4 calculation. Even with age adjustment in regression analysis, residual confounding cannot be completely ruled out. Furthermore, factors unrelated to the degree of fibrosis may have an impact on FIB-4 because it is an indirect marker that includes platelet count and transaminase levels. Therefore, rather than fibrosis that has been histologically verified, our results should be viewed as relationships with FIB-4-defined fibrosis risk.

According to exploratory studies, Group C’s TyG index and FIB-4 had a significant negative connection (ρ = −0.418, *P* = 0.014), which is consistent with the direction of the TFQI-FIB-4 association. However, due to a lack of statistical power, mediation analysis failed to find a significant mediating effect of fasting C-peptide in Group B. Ferritin, liver enzymes, and APRI were among the other exploratory markers that did not consistently or significantly correlate with FIB-4 or TFQI.

There are a few restrictions to be aware of. *Post-hoc* power analysis revealed poor statistical power, especially in Group B (0.40), and the sample size was small (34 patients per group). Although age-stratified analysis was taken into consideration, it was not carried out because there would not have been enough statistical power (about 17 patients per subgroup) to find significant correlations if 34 patients had been divided into age subgroups. Selection bias could be introduced by the single-center retrospective design. Our findings represent predicted fibrosis risk rather than histologically verified fibrosis severity because FIB-4 is used as a surrogate measure. Regression models may account for the age disparity between groups, but residual confounding may still be introduced. It is impossible to completely rule out the possibility of false-positive results due to the small sample size and exploratory nature of many investigations. Therefore, to confirm these preliminary results, larger prospective cohorts with direct fibrosis assessments are required.

This study has a number of advantages despite these drawbacks. To the best of our knowledge, this is the first study to explicitly compare the TFQI-FIB-4 association in three different metabolic backgrounds using a single study design: MASLD alone, MASLD with T2DM, and MASLD with T2DM with treated PH. Information on the thyroid-liver axis under clinically optimal thyroid function, which has seldom been investigated in prior studies, is provided by the inclusion of PH patients who had met treatment goals. A clear framework for assessing the robustness of the results is provided by the methodical approach to differentiating primary, sensitivity, and exploratory analyses. The observed heterogeneity across metabolic backgrounds may help guide the design of future hypothesis-driven studies, even though our results are exploratory and need validation.

## Data Availability

The raw data supporting the conclusions of this article will be made available by the authors, without undue reservation.

## References

[1] Younossi ZM Golabi P Paik JM Henry A Van Dongen C Henry L . The global epidemiology of nonalcoholic fatty liver disease (NAFLD) and nonalcoholic steatohepatitis (NASH): a systematic review. Hepatology. (2023) 77:1335–47. doi: 10.1097/hep.0000000000000004 36626630 PMC10026948

[2] Chan KE Ong EYH Chung CH Ong CEY Koh B Tan DJH . Longitudinal outcomes associated with metabolic dysfunction-associated steatotic liver disease: a meta-analysis of 129 studies. Clin Gastroenterol Hepatol. (2024) 22:488–98. doi: 10.1016/j.cgh.2023.09.018 37775028

[3] Targher G Byrne CD Tilg H . MASLD: a systemic metabolic disorder with cardiovascular and Malignant complications. Gut. (2024) 73:691–702. doi: 10.1136/gutjnl-2023-330595 38228377

[4] Younossi ZM Golabi P de Avila L Paik JM Srishord M Fukui N . The global epidemiology of NAFLD and NASH in patients with type 2 diabetes: a systematic review and meta-analysis. J Hepatol. (2019) 71:793–801. doi: 10.1016/j.jhep.2019.06.021 31279902

[5] Chng CL Goh GBB Yen PM . Metabolic and functional cross talk between the thyroid and liver. Thyroid. (2025) 35:607–23. doi: 10.1089/thy.2025.0178 40420529

[6] Cao L An Y Liu H Jiang J Liu W Zhou Y . Global epidemiology of type 2 diabetes in patients with NAFLD or MAFLD: a systematic review and meta-analysis. BMC Med. (2024) 22:101. doi: 10.1186/s12916-024-03315-0 38448943 PMC10919055

[7] Yuan S Ebrahimi F Bergman D Vujković M Scorletti E Ruan X . Thyroid dysfunction in MASLD: results of a nationwide study. JHEP Rep. (2025) 7:101369. doi: 10.1016/j.jhepr.2025.101369 40342634 PMC12060450

[8] Betlejewska J Hubska J Roszkowska Z Maciejczyk A Bachurska D Domański J . Endocrine disorders and metabolic dysfunction-associated steatotic liver disease: a narrative review. Biomedicines. (2025) 13:2500. doi: 10.3390/biomedicines13102500 41153782 PMC12562047

[9] Polyzos SA Targher G . Hepatic thyroid hormone receptor-β signalling: mechanisms and recent advancements in the treatment of metabolic dysfunction-associated steatohepatitis. Diabetes Obes Metab. (2025) 27:1635–47. doi: 10.1111/dom.16117 39658733

[10] Mantovani A Csermely A Bilson J Borella N Enrico S Pecoraro B . Association between primary hypothyroidism and metabolic dysfunction-associated steatotic liver disease: an updated meta-analysis. Gut. (2024) 73:1554–61. doi: 10.1136/gutjnl-2024-332491 38782564

[11] Amdetsion GY Pan CW Tebeje H Sapkota A Nandyal S Kotwal V . Association between subclinical hypothyroidism and MASLD: a systematic review and meta-analysis. Int J Hepatol. (2025) 2025:8133686. doi: 10.1155/ijh/8133686 41357781 PMC12681400

[12] Maurotti S Scopacasa B Scionti F Pujia R Frosina M Mirarchi A . Raman spectroscopic characterization of liver steatosis and fibrosis in a 2D and 3D *in vitro* thyroxine-treated hypothyroid cellular model. Mol Cell Endocrinol. (2026) 611:112679. doi: 10.1016/j.mce.2025.112679 41109556

[13] Casula S Ettleson MD Bianco AC . Are we restoring thyroid hormone signaling in levothyroxine-treated patients with residual symptoms of hypothyroidism? Endocr Pract. (2023) 29:581–8. doi: 10.1016/j.eprac.2023.04.003 37419565 PMC11221272

[14] Laclaustra M Moreno-Franco B Lou-Bonafonte JM Mateo-Gallego R Casasnovas JA Guallar-Castillon P . Impaired sensitivity to thyroid hormones is associated with diabetes and metabolic syndrome. Diabetes Care. (2019) 42:303–10. doi: 10.2337/dc18-1410 30552134

[15] Wang Z Yu H Wang K Han J Song Y . Association between thyroid hormone resistance and obesity: a cross-sectional study and mouse stimulation test. Obes (Silver Spring). (2024) 32:1483–93. doi: 10.1002/oby.24084 39045674

[16] Yao X Xiao K Oo HK . Impaired thyroid hormone sensitivity is associated with increased risk of hepatic fibrosis in euthyroid population: a cross-sectional analysis of NHANES. Horm Metab Res. (2025) 57:511–9. doi: 10.1055/a-2700-6797 40935129

[17] Rinella ME Lazarus JV Ratziu V Francque SM Sanyal AJ Kanwal F . A multisociety Delphi consensus statement on new fatty liver disease nomenclature. J Hepatol. (2023) 79:1542–56. doi: 10.1097/hep.0000000000000520 37364790

[18] American Diabetes Association Professional Practice Committee for Diabetes* . 2. Diagnosis and classification of diabetes: standards of care in diabetes-2026. Diabetes Care. (2026) 49:S27–49. doi: 10.2337/dc26-S002 PMC12690183 41358893

[19] European Association for the Study of the Liver (EASL) European Association for the Study of Diabetes (EASD) European Association for the Study of Obesity (EASO) . EASL-EASD-EASO clinical practice guidelines on the management of metabolic dysfunction-associated steatotic liver disease (MASLD). Obes Facts. (2024) 17:374–444. doi: 10.1159/000539371 38852583 PMC11299976

[20] Lee J Vali Y Boursier J Spijker R Anstee QM Bossuyt PM . Prognostic accuracy of FIB-4, NAFLD fibrosis score and APRI for NAFLD-related events: a systematic review. Liver Int. (2021) 41:261–70. doi: 10.1111/liv.14669 32946642 PMC7898346

[21] Chalasani N Younossi Z Lavine JE Charlton M Cusi K Rinella M . The diagnosis and management of nonalcoholic fatty liver disease: practice guidance from the American Association for the Study of Liver Diseases. Hepatology. (2018) 67:328–57. doi: 10.1002/hep.29367 28714183

[22] Guerrero-Romero F Simental-Mendía LE González-Ortiz M Martínez-Abundis E Ramos-Zavala MG Hernández-González SO . The product of triglycerides and glucose, a simple measure of insulin sensitivity. Comparison with the euglycemic-hyperinsulinemic clamp. J Clin Endocrinol Metab. (2010) 95:3347–51. doi: 10.1210/jc.2010-0288 20484475

[23] Li Y Wang F . Impaired thyroid hormone sensitivity is associated with the degrees of fatty infiltration in metabolic dysfunction associated steatotic liver disease. Front Endocrinol (Lausanne). (2025) 16:1646790. doi: 10.3389/fendo.2025.1646790 41048433 PMC12490986

[24] Zhang X Liu J Wang Q Han C Yan Y Xiang X . Impaired sensitivity to thyroid hormone is associated with developing non-alcoholic fatty liver disease in euthyroid diabetic subjects. Front Endocrinol (Lausanne). (2024) 15:1450049. doi: 10.3389/fendo.2024.1450049 39744179 PMC11688208

[25] Mostafa AM Fouad Y Gaber Y Alem SA Pan Z Eslam M . Differential risk factors of fibrosis between lean and obese MAFLD. Clin Exp Med. (2025) 25:211. doi: 10.1007/s10238-025-01749-1 40531393 PMC12177025

